# SpectroFood dataset: A comprehensive fruit and vegetable hyperspectral meta-dataset for dry matter estimation

**DOI:** 10.1016/j.dib.2024.110040

**Published:** 2024-01-11

**Authors:** Ioannis Malounas, Wout Vierbergen, Sezer Kutluk, Manuela Zude-Sasse, Kai Yang, Ming Zhao, Dimitrios Argyropoulos, Jonathan Van Beek, Eva Ampe, Spyros Fountas

**Affiliations:** a Agricultural University of Athens (AUA), Iera Odos 75, 11855 Athens, Greece; b Technology and Food Science Unit, Flanders Research Institute for Agriculture, Fisheries and Food (ILVO), Burgemeester Van Gansberghelaan 115 bus 1, 9820 Merelbeke, Belgium; c Department of Datascience in Bioeconomy, Leibniz Institute for Agricultural Engineering and Bioeconomy (ATB), Max-Eyth-Allee 100, 14469 Potsdam-Bornim, Germany; d Department of Agromechatronic, Leibniz Institute for Agricultural Engineering and Bioeconomy (ATB), Max-Eyth-Allee 100, 14469 Potsdam-Bornim, Germany; e School of Biosystems and Food Engineering, University College Dublin (UCD), Stillorgan Rd, Belfield, Dublin 4, Ireland; f INAGRO VZW, Ieperseweg 87, 8800 Rumbeke-Beitem, Belgium

**Keywords:** Hyperspectral imaging, Artificial intelligence, Apple, Broccoli, Leek, Mushroom

## Abstract

In the dataset presented in this article, samples belonging to one of the following crops, apple, broccoli, leek, and mushroom, were measured by hyperspectral cameras in the visible/near-infrared spectral domain (430-900 nm). The dataset was compiled by putting together measurements from different calibrated hyperspectral imaging cameras and crops to facilitate the training of artificial intelligence models, helping to overcome the generalization problem of hyperspectral models. In particular, this dataset focuses on estimating dry matter content across various crops by a single model in a non-destructive way using hyperspectral measurements. This dataset contains extracted mean reflectance spectra for each sample (n=1028) and their respective dry matter content (%).

Specifications Table

**Table:** 

Subject	Agriculture Engineering
Specific subject area	Hyperspectral imaging and artificial intelligence for the non-destructive estimation of fruit and vegetable quality characteristics
Data format	Analyzed
Type of data	Table, Hyperspectral image
Data collection	Data were acquired using four hyperspectral imaging cameras. Imec Snapscan for broccoli, SpecimIQ for mushroom, Specim FX10 and FX17 for leek and Cubert ULTRIS S20 for apple. For all measurements, the following protocol was followed: 1. Hyperspectral imaging mode: Reflectance 2. Lighting system: stabilized halogen lamps with good performance in the wavelength range of 400-1000 nm 4. The measurement distance between the imaging unit and sample was fixed for each crop. 5. Dry matter content measurement method: Dry using a convective air dryer until reaching a constant mass. All samples were selected to have a reasonable variation in dry matter. Finally, the following formula was used for reflectance calibration: $Rc=\frac{Ro-D}{W-D}\;\times \;100$ Ro: raw hyperspectral image, W: image of a reference object of uniform, stable, and high reflectance standard (∼100% reflectance), D: dark image (∼0% reflectance), Rc: corrected hyperspectral image.
Data source location	Data are stored at Agricultural University of Athens (AUA) premises. Iera Odos 75, 11855 Athens, Greece, Department of Horticultural Engineering
Data accessibility	Repository name:Zenodo Table data Data identification number: 10.5281/zenodo.8362947 Direct URL to data: https://zenodo.org/record/8362947 Hyperspectral image data 1) Data identification number: 10.5281/zenodo.10301753 Direct URL to data: https://zenodo.org/records/10301753 2) Data identification number: 10.5281/zenodo.10302438 Direct URL to data: https://zenodo.org/records/10302438 3) Data identification number: 10.5281/zenodo.10302426 Direct URL to data: https://zenodo.org/records/10302426 4) Data identification number: 10.5281/zenodo.10302386 Direct URL to data: https://zenodo.org/records/10302386

## 1 Value of the Data

• Spectra were acquired using calibrated hyperspectral imaging systems under the same controlled conditions for four crops with high variation of dry matter values amongst the same crop and across all four.
• The dry matter content of the four crops is the common variable when considering the quality of the fresh produce.
• This dataset can be used to try novel data processing approaches to solve one of the most prominent problems of hyperspectral imaging models. Namely, the ability of models to generalize across different sensors and crops
• The agrifood industry and researchers can use this dataset to build models to predict the dry matter content of apples, broccoli, leek, and mushrooms or use it as validation towards robust indicators for other crops.
• This dataset can serve as a test dataset for scientists to perform future experiments, benchmark their solutions, and create new agrifood meta-datasets.

## 2 Background

Hyperspectral imaging is widely used and studied for non-destructive determination of quality characteristics in fruits and vegetables. However, despite the extended research, the majority of models trained to estimate or classify samples are not evaluated outside their training and validation dataset. Furthermore, all those models cannot be generalized to other types of crops or are not compatible with other brands of sensors. The SpectroFood dataset aims to be used as a benchmarking tool for Artificial Intelligence models and as a dataset to test novel techniques capable of a higher degree of generalization.

## 3 Data Description

The SpectroFood dataset [1], SpectroFood dataset Apple [2], SpectroFood dataset Broccoli [3], SpectroFood dataset Leek [4] and SpectroFood dataset Mushroom [5] as well as the respective files they contain: SpectroFood_dataset.csv, Apple.mat, Broccoli_1-Broccoli_25.mat, Leek_1-Leek_9.mat and Mushroom_1-Mushroom_5.mat are comprised of chemical and VIS-NIR measurements captured across four crops and using four cameras (Table 1). The csv file contains the extracted spectral data from all crops in tabular form as well as the dry matter measurements while the mat files the hyperspectral data for each of the crops, as their name suggets.

**Table 1:** Camera used for each crop and distance between sample and camera.

Crop	Apple	Broccoli	Leek	Mushroom
Camera	Cubert ULTRIS S20	Imec Snapscan	Specim FX10 and FX17	SpecimIQ
Distance between sample and camera	50cm	30cm	60cm	25cm

In total, 1028 samples were measured across a spectral range of 398 to 1717 nm, with all measurements capturing the VIS-NIR range of 470-900 nm (Table 2).

**Table 2:** Number of measurements per crop and their spectral details.

	Apple	Broccoli	Leek	Mushroom
Number of measurements	240	250	288	250
Spectral range	430-990nm	470-900nm	Specim FX10 (398 to 931 nm) and FX17 (936 to 1717 nm)	400-998nm
Spectral resolution/No. of bands	141	150	421	204
Full width half maximum (FWHM).	12nm	10-15nm	Specim FX10 (2.62-2.82 nm) and FX17 (3.46-3.58 nm)	7nm

**Fig. 1:**
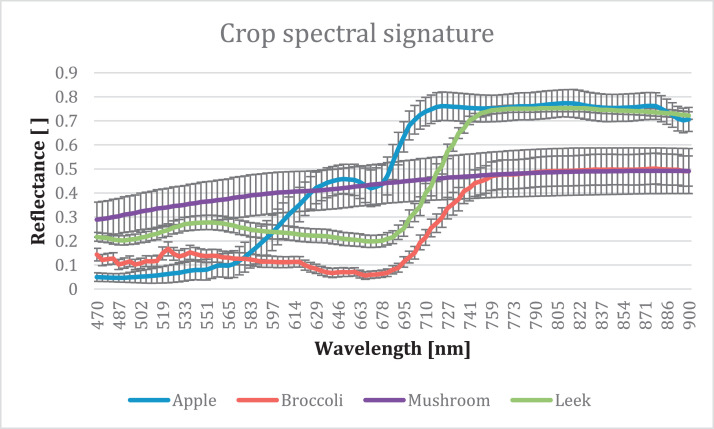
Crop spectral signatures based on averaged values with their standard deviation.

The dataset is structured so that the rows correspond to the samples measured and the columns to the variables measured. The first column contains the sample number and the crop, the second column contains the dry matter content, and the following columns contain the reflectance spectra obtained for each specified spectral wavelength. The mean spectrum for all crops can be seen in Fig. 1.

Finally, the dry matter content across measurements ranges from 8.1% to 87% (Table 2)

## 4 Experimental Design, Materials and Methods

### 4.1 Data

#### 4.1.1 Hyperspectral image acquisition

Reflectance spectra were acquired for all four crops by placing the samples in an illumination-controlled environment to maximize the dynamic range of the sensors for each sample. Across all use cases, the same acquisition protocol was followed to ensure the consistency of all measurements. The protocol consisted of the following actions: i) set the hyperspectral imaging mode to Reflectance, ii) capture white and dark references using a stable and high reflectance standard (∼100% reflectance) and a dark image (∼0% reflectance), respectively, iii) use of halogen lamps (Apple: 150W from Illumination Technologies; Broccoli and Leek: 50W from Osram; Mushroom: 245W from Specim) with good performance at the wavelength range of 400-1000 nm, together with a stabilized DC power supply to stabilize them, iii) maintain a constant distance between sensor and sample throughout each measurement campaign. The distance was kept the same across each imaging campaign; however, it was different for each crop and camera setup to optimize data quality based on the specific characteristics of each camera (e.g., linescan, snapscan) and crop (e.g., shape). Finally, samples were placed in the image acquisition stage once the setup was ready for capturing to minimize sample exposure to the heat produced by the halogen lamps.

### 4.2 Dry matter measurements

Once the spectral measurements were completed, the samples were immediately processed for dry matter measurements. All samples were weighed to measure their fresh weight and then placed in trays and transported to a convective air dryer (oven) until they reached a constant mass. Based on the fresh weight of each sample and their final constant mass (final weight), their moisture content was calculated using the following equation.

$$Moisture\;content\;\left(\%\right)=\frac{\text{Fresh}\;\text{weight}-\text{Final}\;\text{weight}}{\text{Fresh}\;\text{weight}}x100$$

One moisture content value (% wet basis (w.b.)) was recorded for each sample, and then the dry matter was calculated by subtracting moisture percentage (%) from 100%.

### 4.3 Data transformations

The dataset does not consist of the raw hyperspectral imaging data. Instead, all captured hyperspectral images have been automatically radiometrically corrected by the image acquisition software provided by each hyperspectral camera manufacturer. After data acquisition, the reflectance calibration was performed on the acquired data using the calibration function below:

$$Rc=\frac{Ro-D}{W-D}x\;100$$

where Ro is the raw hyperspectral image, W is the image of a reference object of uniform, stable, and high reflectance standard (∼100% reflectance), D is the dark image (∼0% reflectance), and Rc is the corrected hyperspectral image.

For obtaining the extracted spectra, dead pixels and spikes were removed using fixed thresholding values while background was removed either through manual segmentation or using background removal algorithms such as the Otsu algorithm. However, to allow for better model generalisation, additional preprocessing (provisions) may be needed depending on the specific use case to address the differences between each crop, the size, and light penetration properties.

## Limitations

Despite all cameras being manufactured by scientifically credible companies, measurements may differ due to the different sensors used. Image quality due to sensor noise and sensitivity must be considered when handling the data. Moreover, due to the relatively small number of samples for each crop, parties interested in applying machine learning models should pay attention to avoid data overfitting (Table 3).

**Table 3:** Dry matter content (DMC) per crop.

DMC in %	Apple	Broccoli	Leek	Mushroom
Min	14 %	12%	8.1%	5%
Max	17 %	20%	19%	87%
Average	15 %	15%	12%	29%

## Ethics Statements

The authors affirm that no human or animal experiments were conducted nor social media data was collected.

## CRediT authorship contribution statement

**Ioannis Malounas:** Methodology, Investigation, Data curation, Writing – original draft. **Wout Vierbergen:** Methodology, Investigation. **Sezer Kutluk:** Methodology. **Manuela Zude-Sasse:** Conceptualization, Methodology, Investigation, Writing – review & editing, Funding acquisition. **Kai Yang:** Methodology, Investigation. **Ming Zhao:** Methodology, Investigation. **Dimitrios Argyropoulos:** Conceptualization, Methodology, Investigation, Writing – review & editing, Funding acquisition. **Jonathan Van Beek:** Conceptualization, Methodology, Investigation, Writing – review & editing, Funding acquisition. **Eva Ampe:** Methodology, Investigation. **Spyros Fountas:** Conceptualization, Methodology, Writing – original draft, Supervision, Project administration, Funding acquisition.

## Data Availability

SpectroFood dataset Apple (Original data) (Zenodo) SpectroFood dataset (Original data) (ZENODO) SpectroFood dataset Leek (Original data) (Zenodo) SpectroFood dataset Mushroom (Original data) (Zenodo) SpectroFood dataset Broccoli (Original data) (Zenodo).
